# Crystal structure of methyl 1,3-benzoxazole-2-carboxyl­ate

**DOI:** 10.1107/S2056989021010094

**Published:** 2021-10-08

**Authors:** Alexandre Poirot, Nathalie Saffon-Merceron, Nadine Leygue, Eric Benoist, Suzanne Fery-Forgues

**Affiliations:** a Université de Toulouse III Paul Sabatier, Laboratoire SPCMIB, UMR CNRS 5068, 118 route de Narbonne, F-31062 Toulouse, France; b Université de Toulouse III Paul Sabatier, Institut de Chimie de Toulouse, ICT-UAR 2599, 118, route de Narbonne, F-31062 Toulouse, France

**Keywords:** crystal structure, benzoxazole, herringbone arrangement, γ packing type, π–π inter­actions, strong C—H⋯N hydrogen bonds

## Abstract

The title compound, C_9_H_7_NO_3_, crystallizes in the monoclinic (*P*2_1_) space group. In the crystal, the almost planar mol­ecules display a flattened herringbone arrangement. Stacking mol­ecules are slipped in the lengthwise and widthwise directions and are linked by π–π inter­actions [*d*(*Cg*⋯*Cg* = 3.6640 (11) Å]. The structure is characterized by strong C—H⋯N and weak C—H⋯O hydrogen bonds, and further stabilized by C–O⋯π inter­actions.

## Abstract 2

The herringbone structure of methyl 1,3-benzoxazole-2-carboxyl­ate is characterized by strong C—H⋯N and weak C—H⋯O hydrogen bonds, and further stabilized by C—O⋯π and π–π inter­actions.

## Chemical context

Benzoxazoles are common in natural products and represent an important class of key structural motifs, often incorporated as building blocks in ligands to target a variety of receptors and enzymes in medicinal chemistry studies (Demmer & Bunch, 2015 ▸; Kamal *et al.*, 2020 ▸). They are also a scaffold of prime importance for fluorescent probes and materials (Carayon & Fery-Forgues, 2017 ▸; Fery-Forgues & Vanucci-Bacqué, 2021 ▸). Methyl-1,3-benzoxazole-2-carboxyl­ate (**1**) belongs to this family and much attention has been paid to its preparation. 
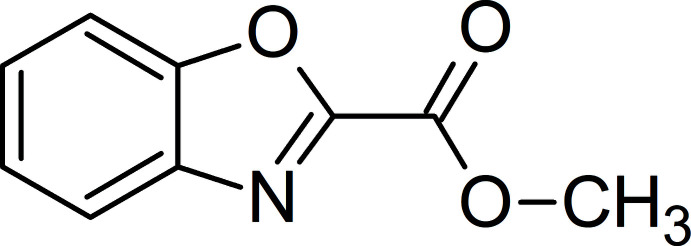


This compound was first prepared by a multi-step synthesis starting from 2,3-dioxo-1,4-benzoxazine (Dickoré *et al.*, 1970 ▸) and 2-cyano­benzoxazole (Möller, 1970 ▸), but it can be obtained much more simply from condensation of 2-amino­phenol with methyl 2,2,2-tri­meth­oxy­acetate (Musser, Hudec *et al.*, 1984 ▸; Koshelev *et al.*, 2019 ▸). It has been synthesized in high yields by direct carboxyl­ation of benzoxazole using carbon dioxide (CO_2_) as a naturally abundant and renewable C1 source, with (Zhang *et al.*, 2010 ▸; Inomata *et al.*, 2012 ▸) or without any metal catalyst (Vechorkin *et al.*, 2010 ▸; Fenner & Ackermann, 2016 ▸). Recently, it has been produced by oxidative cyclization of glycine catalysed by copper (Liu *et al.*, 2021 ▸) or induced by irradiation with visible light (Zhu *et al.*, 2021 ▸). The mol­ecule is commercially available. It has been used to complex europium, resulting in a very efficient electroluminescent layer for applications in the field of organic light-emitting diodes (OLEDs) (Koshelev *et al.*, 2019 ▸). Used as a synthetic inter­mediate, methyl-1,3-benzoxazole-2-carboxyl­ate has led to various pharmacologically active agents with anti-allergic (Musser, Brown *et al.*, 1984 ▸), anti-microbial (Vodela *et al.*, 2013 ▸) and neuro-anti-inflammatory (Shang *et al.*, 2020 ▸) activity, to name just a few.

## Structural commentary

**Figure 1:**
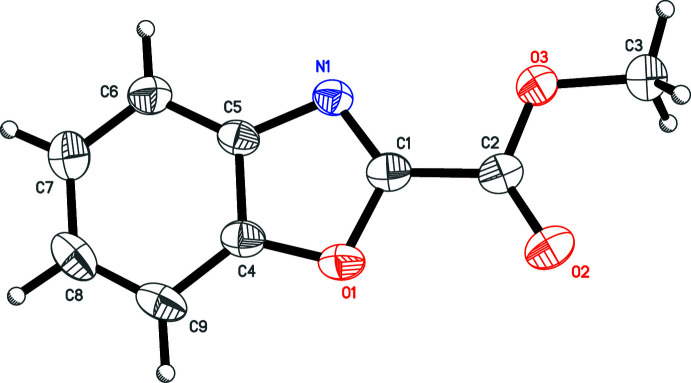
The mol­ecular structure of the title compound with the atom numbering. The displacement ellipsoids are drawn at the 50% probability level.

The title compound (Fig. 1 ▸) crystallizes in the monoclinic space group *P*2_1_ and exhibits the expected bond lengths and angles for a benzoxazole. The N1—C1 bond, which corresponds to a double bond, is significantly shorter [1.293 (2) Å] than the other bonds (>1.36 Å) of the oxazole cycle. The mol­ecule is almost planar [N1—C1—C2—O3 = −6.7 (2)°]. The heterocyclic and carbonyl oxygen atoms O1 aand O2, respectively, are located on the same side with respect to the long axis of the mol­ecule.

## Supra­molecular features

**Figure 2:**
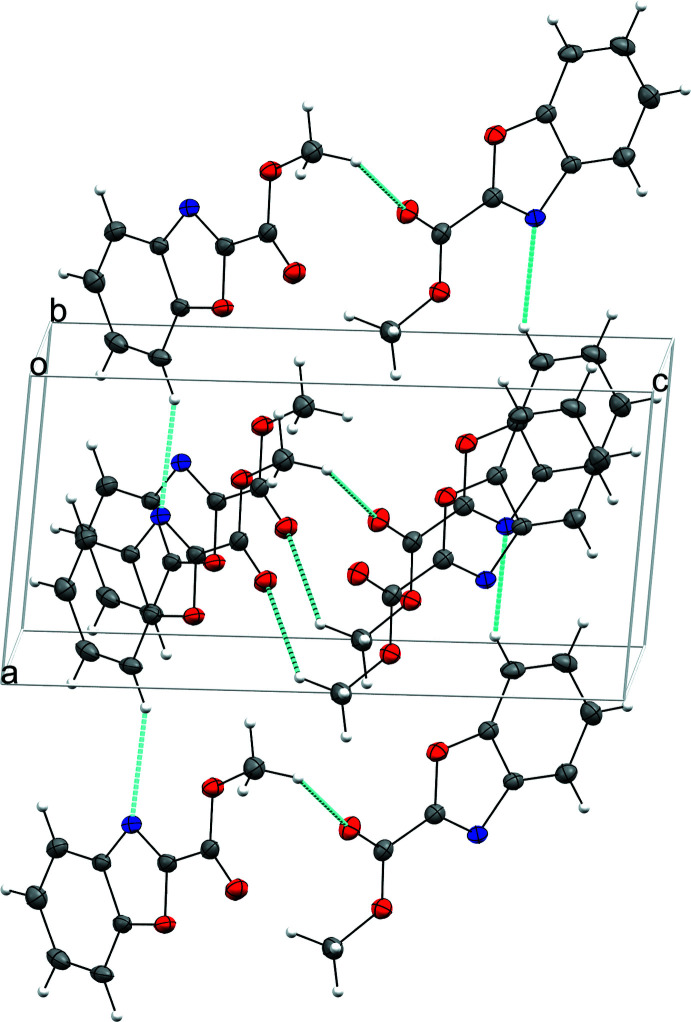
C—H⋯N and C—H⋯O hydrogen bonds (blue dotted lines).

**Figure 3:**
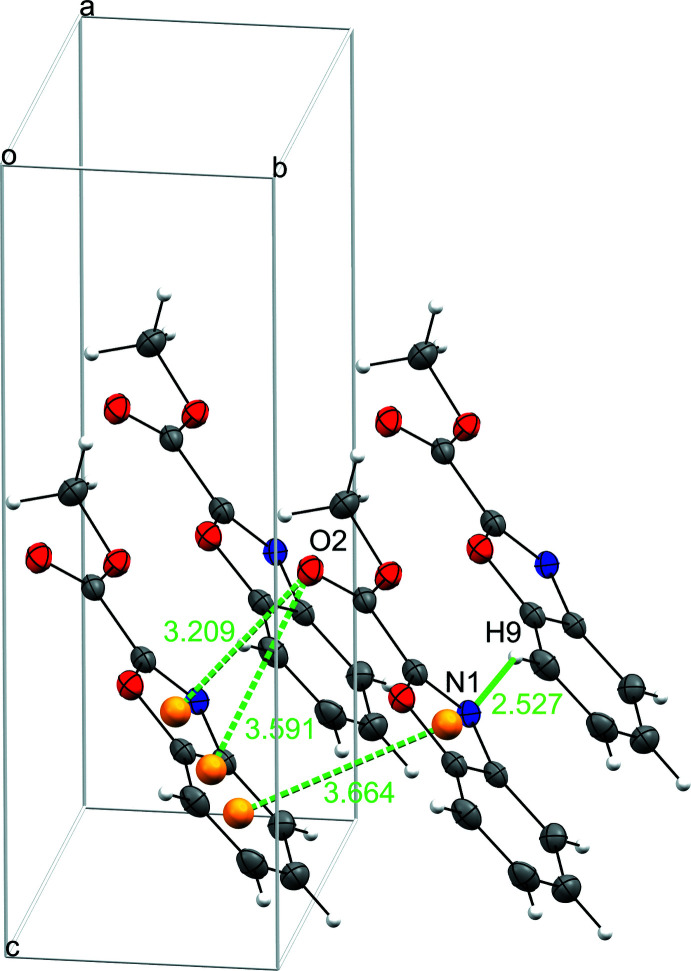
π–π and C—O⋯π inter­actions (green dotted lines). Orange balls represent the ring centroids *Cg*.

In the crystal structure, mol­ecules are displayed according to the γ packing type, *i.e*. a flattened herringbone featuring stacks of parallel, translationally related mol­ecules (Desiraju *et al.*, 1989 ▸; Campbell *et al.*, 2017 ▸) (Fig. 2 ▸). Neighboring mol­ecules situated in almost perpendicular planes (84.4°) are linked through C—H⋯N inter­actions between the heterocyclic nitro­gen atom N1 and H9 of an adjacent mol­ecule and weak C—H⋯O hydrogen bonds between O2 and one hydrogen atom of the methyl group (Table 1 ▸, Fig. 2 ▸). Strong C—O⋯π inter­actions are also important for the stabilization of the structure (Table 2 ▸, Fig. 3 ▸). Stacking mol­ecules are slipped in the lengthwise and widthwise directions and linked by π– π inter­actions [centroid–centroid distance = 3.6640 (11) Å] (Table 3 ▸).

## Database survey

Benzoxazole-based mol­ecules have given an umpteen number of crystal structures. A search of the Cambridge Structural Database (CSD, version of November 2020; Groom *et al.*, 2016 ▸) found only twelve benzoxazoles substituted by a carbonyl group on the 2-position. In almost half of the cases, the benzoxazole derivative is used as a ligand to complex an Ni, Co or Cu atom (CAYSIG and CAYSOM; Iasco *et al.*, 2012 ▸; LAJNAN; Zhang *et al.*, 2010 ▸), or incorporated in a macromolecule (NESPUY; Lim *et al.*, 2012 ▸; LUYJUL; Osowska & Miljanić, 2010 ▸), resulting in a geometry quite far from that of a small entity. Among the remaining examples, the benzoxazolylcarbonyl moiety may be linked to an aromatic group. When the latter is a phenyl group, the mol­ecule is almost planar (ROFZUJ; Boominathan *et al.*, 2014 ▸). With another benzoxazole heterocycle, the dihedral angle is only around 8° (AGESUD; Boga *et al.*, 2018 ▸). In contrast, this angle almost reaches 71° with a benzoic acid that is involved in many inter­molecular inter­actions (DEJGEE; Ling *et al.*, 1999 ▸), and when the benzoxazole and phenyl derivative moieties are attached *via* a flexible linker (KONTEP; Deng *et al.*, 2019 ▸). Finally, the benzoxazolylcarbonyl moiety may be linked to an aliphatic moiety, which may be rather bulky like a bornane-1,2-sultam moiety (BAKRIQ; Piątek *et al.*, 2011 ▸), or smaller like a morpholine moiety (JAXMED; Xing *et al.*, 2017 ▸). In both cases, the network is structured by an interaction between the carbonyl oxygen of one molecule and the hydrogen atom borne by the C7 carbon of a neighbouring molecule. Finally, the framework closest to that of the title compound is an isopropyl 4-acetyl-5-hy­droxy-1,3-benzoxazole-2-carboxyl­ate (MIMZUG; Tangellamudi *et al.*, 2018 ▸). In this mol­ecule, the hydroxyl and the acetyl substituents form intra­molecular hydrogen bonds while the carbonyl oxygen of one mol­ecule inter­acts with the isopropyl group of the neigbouring one to form some kind of dimer. In general, planar mol­ecules tend to assemble in layers (AGESUD; Boga *et al.*, 2018 ▸; MIMZUG; Tangellamudi *et al.*, 2018 ▸) and even in ribbons (JAXMED; Xing *et al.*, 2017 ▸).

## Synthesis and crystallization

**Figure 4:**
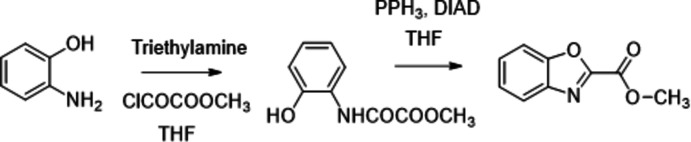
Synthesis route to methyl-1,3-benzoxazole-2-carboxyl­ate.

The title compound was synthesized according to a variant of the procedure described by Jacobs *et al.* (2017 ▸) (Fig. 4 ▸). To a mixture of 5-amino­phenol (1.09 g, 0.01 mol) and tri­ethyl­amine (2.02 g, 0.02 mol) in anhydrous tetra­hydro­furan (40 mL) at 263 K was added slowly methyl oxalyl chloride (1.34 g, 0.011 mol). The mixture was stirred at room temperature for 3 h and then cooled onto an ice–water bath. Tri­phenyl­phosphine (5.64 g, 0.0215 mol), diisopropyl azodi­carboxyl­ate (2.25 g, 0.011 mol) and tetra­hydro­furan (50 mL) were then added. The solution was allowed to stir at room temperature for 16 h and concentrated *in vacuo*. The crude product was purified by column chromatography (SiO_2_, petroleum ether/di­chloro­methane 70/30 *v*/*v* until 60/40 *v*/*v*) to give a white solid (1.2 g) in 83% yield. ^1^H NMR (300 MHz, CDCl_3_): δ = 7.90 (*ddd*, *J* = 7.9, 1.5, 0.8 Hz, 1H), 7.67 (*ddd*, *J* = 8.1, 1.2, 0.8 Hz, 1H), 7.57–7.44 (*m*, 2H), 4.10 (s, 3H). ^13^C NMR (75 MHz, CDCl_3_): δ = 156.9, 152.5, 150.9, 140.5, 128.2, 125.8, 122.2, 111.7, 53.7.

Single crystals of the title compound, suitable for X-ray analysis, were grown by slow evaporation of a di­chloro­methane solution.

## Refinement

Crystal data, data collection and structure refinement details are summarized in Table 4 ▸. All H atoms were fixed geometrically and treated as riding atoms with C—H = 0.95 Å (aromatic) or 0.98 Å (CH_3_), with *U*_iso_(H) = 1.2*U*_eq_(C) or 1.5*U*_eq_(CH_3_).

## Supplementary Material

Crystal structure: contains datablock(s) I. DOI: 10.1107/S2056989021010094/dj2033sup1.cif

Structure factors: contains datablock(s) I. DOI: 10.1107/S2056989021010094/dj2033Isup3.hkl

Supporting information file. DOI: 10.1107/S2056989021010094/dj2033Isup4.cml

CCDC reference: 2112709

Additional supporting information:  crystallographic information; 3D view; checkCIF report

## Figures and Tables

**Table 1:** Hydrogen-bond geometry (Å, °)

*D*—H⋯*A*	*D*—H	H⋯*A*	*D*⋯*A*	*D*—H⋯*A*
C9—H9⋯N1^i^	0.95	2.53	3.377 (2)	149
C3—H3*C*⋯O2^ii^	0.98	2.65	3.389 (2)	133

Symmetry codes: (i) x-1, y, z; (ii) -x+1, y+{\script{1\over 2}}, -z+1.

**Table 2:** C—O⋯π inter­actions (Å, °) *Cg*1 is the centroid of the O1/C1/N1/C5/C4 ring and *Cg*3 is the centroid of the O1/C1/C5–C9 ring.

*X*	*I*	*J*	*I*⋯*J*	*X*⋯*J*	*X*—*I*⋯*J*
C2	O2	*Cg*1^ii^	3.2088 (14)	3.5487 (18)	96.39 (10)
C2	O2	*Cg*3^ii^	3.5912 (14)	3.7321 (17)	87.29 (10)

Symmetry code: (ii) *x*, −1 + *y*, *z*.

**Table 3:** π–π inter­action (Å, °) *Cg*1 is the centroid of the O1/C1/N1/C5/C4 ring and *Cg*2 is the centroid of the C4–C9 ring. *CgI*⋯*CgJ* is the distance between ring centroids. α is the dihedral angle between the planes of the rings *I* and *J*. *CgI*_perp_ and *CgJ*_perp_ are the perpendicular distances of *CgI* from ring *J* and of *CgJ* from ring *I*, respectively. *CgI*_Offset_ and *CgJ*_Offset_ are the distances between *CgI* and the perpendicular projection of *CgJ* on ring *I*, and between *CgJ* and the perpendicular projection of *CgI* on ring *J*, respectively.

*I*	*J*	*CgI*⋯*CgJ*	α	*CgI*_perp_	*CgJ*_perp_	*CgI*_Offset_	*CgJ*_Offset_
1	2^ii^	3.6640 (11)	0.19 (9)	3.3115 (7)	3.3065 (8)	1.579	1.568

Symmetry code: (ii) *x*, −1 + *y*, *z*.

**Table 4:** Experimental details

Crystal data	
Chemical formula	C_9_H_7_NO_3_
*M*_r_	177.16
Crystal system, space group	Monoclinic, *P*2_1_
Temperature (K)	193
*a*, *b*, *c* (Å)	6.8165 (3), 4.4676 (2), 13.2879 (6)
β (°)	95.1319 (16)
*V* (Å^3^)	403.04 (3)
*Z*	2
Radiation type	Mo *K*α
μ (mm^−1^)	0.11
Crystal size (mm)	0.40 × 0.30 × 0.10

Data collection	
Diffractometer	Bruker D8-Venture Photon III detector
Absorption correction	Multi-scan (*SADABS*; Krause *et al.*, 2015 ▸)
*T*_min_, *T*_max_	0.698, 0.746
No. of measured, independent and observed [*I* > 2σ(*I*)] reflections	9084, 1954, 1860
*R*_int_	0.022
(sin θ/λ)_max_ (Å^−1^)	0.667

Refinement	
*R*[*F* ^2^ > 2σ(*F* ^2^)], *wR*(*F* ^2^), *S*	0.030, 0.077, 1.10
No. of reflections	1954
No. of parameters	119
No. of restraints	1
H-atom treatment	H-atom parameters constrained
Δρ_max_, Δρ_min_ (e Å^−3^)	0.20, −0.16

Computer programs: *APEX3* and *SAINT* (Bruker, 2018 ▸), *SHELXT* (Sheldrick, 2015*a* ▸), *SHELXL2018/3* (Sheldrick, 2015*b* ▸), *SHELXTL* (Sheldrick, 2008 ▸), *Mercury* (Macrae *et al.*, 2020 ▸), *PLATON* (Spek 2020 ▸) and *publCIF* (Westrip 2010 ▸).

## supplementary crystallographic information

### Crystal data

**Table:**

C_9_H_7_NO_3_	*F*(000) = 184
*M**_r_* = 177.16	*D*_x_ = 1.460 Mg m^−^^3^
Monoclinic, *P*2_1_	Mo *K*α radiation, λ = 0.71073 Å
*a* = 6.8165 (3) Å	Cell parameters from 6695 reflections
*b* = 4.4676 (2) Å	θ = 3.3–28.2°
*c* = 13.2879 (6) Å	µ = 0.11 mm^−^^1^
β = 95.1319 (16)°	*T* = 193 K
*V* = 403.04 (3) Å^3^	Plate, colourless
*Z* = 2	0.40 × 0.30 × 0.10 mm

### Data collection

**Table:**

Bruker D8-Venture Photon III detector diffractometer	1860 reflections with *I* > 2σ(*I*)
Radiation source: Fine-focus sealed tube	*R*_int_ = 0.022
Phi and ω scans	θ_max_ = 28.3°, θ_min_ = 3.3°
Absorption correction: multi-scan (SADABS; Krause *et al.*, 2015)	*h* = −8→9
*T*_min_ = 0.698, *T*_max_ = 0.746	*k* = −5→5
9084 measured reflections	*l* = −17→17
1954 independent reflections	

### Refinement

**Table:**

Refinement on *F*^2^	Primary atom site location: dual
Least-squares matrix: full	Hydrogen site location: inferred from neighbouring sites
*R*[*F*^2^ > 2σ(*F*^2^)] = 0.030	H-atom parameters constrained
*wR*(*F*^2^) = 0.077	*w* = 1/[σ^2^(*F*_o_^2^) + (0.0432*P*)^2^ + 0.0403*P*] where *P* = (*F*_o_^2^ + 2*F*_c_^2^)/3
*S* = 1.10	(Δ/σ)_max_ < 0.001
1954 reflections	Δρ_max_ = 0.20 e Å^−^^3^
119 parameters	Δρ_min_ = −0.16 e Å^−^^3^
1 restraint	

### Special details

**Table:**

Geometry. All esds (except the esd in the dihedral angle between two l.s. planes) are estimated using the full covariance matrix. The cell esds are taken into account individually in the estimation of esds in distances, angles and torsion angles; correlations between esds in cell parameters are only used when they are defined by crystal symmetry. An approximate (isotropic) treatment of cell esds is used for estimating esds involving l.s. planes.

### Fractional atomic coordinates and isotropic or equivalent isotropic displacement parameters (Å^2^)

**Table:**

	*x*	*y*	*z*	*U*_iso_*/*U*_eq_	
O1	0.25255 (16)	0.3885 (3)	0.69933 (9)	0.0329 (3)	
O2	0.43981 (19)	−0.0004 (3)	0.57993 (9)	0.0382 (3)	
O3	0.73220 (17)	0.2017 (3)	0.63833 (9)	0.0341 (3)	
N1	0.54541 (19)	0.5493 (3)	0.77177 (10)	0.0277 (3)	
C1	0.4528 (2)	0.3789 (4)	0.70473 (12)	0.0296 (3)	
C2	0.5378 (2)	0.1703 (4)	0.63294 (12)	0.0297 (3)	
C3	0.8319 (3)	0.0125 (4)	0.57015 (13)	0.0381 (4)	
H3A	0.794777	−0.196824	0.579791	0.057*	
H3B	0.974729	0.034297	0.584461	0.057*	
H3C	0.793506	0.072127	0.500188	0.057*	
C4	0.2149 (2)	0.5919 (4)	0.77264 (12)	0.0291 (3)	
C5	0.3947 (2)	0.6919 (4)	0.81779 (11)	0.0273 (3)	
C6	0.4014 (2)	0.9009 (4)	0.89607 (13)	0.0347 (4)	
H6	0.522730	0.971603	0.928121	0.042*	
C7	0.2220 (3)	0.9998 (4)	0.92465 (14)	0.0401 (4)	
H7	0.220300	1.141802	0.977785	0.048*	
C8	0.0429 (3)	0.8959 (5)	0.87719 (15)	0.0406 (4)	
H8	−0.076652	0.971435	0.898830	0.049*	
C9	0.0342 (2)	0.6878 (5)	0.80018 (14)	0.0370 (4)	
H9	−0.087048	0.615698	0.768384	0.044*	

### Atomic displacement parameters (Å^2^)

**Table:**

	*U*^11^	*U*^22^	*U*^33^	*U*^12^	*U*^13^	*U*^23^
O1	0.0259 (5)	0.0362 (6)	0.0359 (6)	−0.0064 (5)	−0.0013 (4)	0.0001 (5)
O2	0.0406 (6)	0.0349 (6)	0.0380 (6)	−0.0087 (5)	−0.0024 (5)	−0.0035 (5)
O3	0.0315 (6)	0.0342 (6)	0.0364 (6)	−0.0025 (5)	0.0009 (4)	−0.0061 (5)
N1	0.0241 (6)	0.0274 (6)	0.0312 (6)	−0.0017 (5)	0.0008 (4)	0.0002 (5)
C1	0.0288 (7)	0.0286 (7)	0.0311 (7)	−0.0048 (6)	0.0007 (6)	0.0048 (6)
C2	0.0329 (8)	0.0264 (7)	0.0292 (7)	−0.0042 (6)	−0.0006 (6)	0.0039 (6)
C3	0.0384 (9)	0.0380 (10)	0.0381 (8)	0.0030 (8)	0.0047 (7)	−0.0042 (8)
C4	0.0259 (7)	0.0305 (8)	0.0308 (7)	−0.0049 (6)	0.0010 (5)	0.0063 (6)
C5	0.0233 (7)	0.0278 (7)	0.0306 (7)	−0.0019 (6)	0.0013 (5)	0.0057 (6)
C6	0.0311 (8)	0.0357 (8)	0.0366 (8)	−0.0030 (7)	−0.0006 (6)	−0.0014 (7)
C7	0.0438 (10)	0.0370 (9)	0.0407 (9)	0.0020 (8)	0.0096 (7)	−0.0015 (8)
C8	0.0302 (8)	0.0431 (10)	0.0505 (10)	0.0040 (8)	0.0146 (7)	0.0107 (9)
C9	0.0220 (7)	0.0429 (9)	0.0461 (9)	−0.0035 (7)	0.0032 (6)	0.0104 (8)

### Geometric parameters (Å, º)

**Table:**

O1—C1	1.3610 (19)	C4—C9	1.384 (2)
O1—C4	1.373 (2)	C4—C5	1.390 (2)
O2—C2	1.200 (2)	C5—C6	1.395 (2)
O3—C2	1.3281 (19)	C6—C7	1.385 (3)
O3—C3	1.452 (2)	C6—H6	0.9500
N1—C1	1.293 (2)	C7—C8	1.402 (3)
N1—C5	1.395 (2)	C7—H7	0.9500
C1—C2	1.488 (2)	C8—C9	1.380 (3)
C3—H3A	0.9800	C8—H8	0.9500
C3—H3B	0.9800	C9—H9	0.9500
C3—H3C	0.9800		
			
C1—O1—C4	103.51 (12)	C9—C4—C5	123.92 (17)
C2—O3—C3	115.17 (14)	C4—C5—N1	108.64 (15)
C1—N1—C5	103.74 (13)	C4—C5—C6	120.36 (15)
N1—C1—O1	116.33 (15)	N1—C5—C6	131.00 (14)
N1—C1—C2	128.06 (14)	C7—C6—C5	116.58 (16)
O1—C1—C2	115.61 (13)	C7—C6—H6	121.7
O2—C2—O3	126.82 (17)	C5—C6—H6	121.7
O2—C2—C1	123.11 (16)	C6—C7—C8	121.71 (18)
O3—C2—C1	110.07 (14)	C6—C7—H7	119.1
O3—C3—H3A	109.5	C8—C7—H7	119.1
O3—C3—H3B	109.5	C9—C8—C7	122.32 (17)
H3A—C3—H3B	109.5	C9—C8—H8	118.8
O3—C3—H3C	109.5	C7—C8—H8	118.8
H3A—C3—H3C	109.5	C8—C9—C4	115.10 (16)
H3B—C3—H3C	109.5	C8—C9—H9	122.4
O1—C4—C9	128.30 (15)	C4—C9—H9	122.4
O1—C4—C5	107.78 (14)		
			
C5—N1—C1—O1	0.05 (19)	C9—C4—C5—N1	179.93 (15)
C5—N1—C1—C2	−179.34 (15)	O1—C4—C5—C6	−179.61 (14)
C4—O1—C1—N1	0.03 (18)	C9—C4—C5—C6	0.2 (2)
C4—O1—C1—C2	179.50 (13)	C1—N1—C5—C4	−0.11 (17)
C3—O3—C2—O2	1.7 (2)	C1—N1—C5—C6	179.60 (17)
C3—O3—C2—C1	−178.99 (13)	C4—C5—C6—C7	−0.2 (2)
N1—C1—C2—O2	172.61 (18)	N1—C5—C6—C7	−179.92 (16)
O1—C1—C2—O2	−6.8 (2)	C5—C6—C7—C8	−0.1 (3)
N1—C1—C2—O3	−6.7 (2)	C6—C7—C8—C9	0.6 (3)
O1—C1—C2—O3	173.92 (14)	C7—C8—C9—C4	−0.6 (3)
C1—O1—C4—C9	−179.89 (17)	O1—C4—C9—C8	180.00 (16)
C1—O1—C4—C5	−0.10 (16)	C5—C4—C9—C8	0.2 (3)
O1—C4—C5—N1	0.14 (17)		

### Hydrogen-bond geometry (Å, º)

**Table:**

*D*—H···*A*	*D*—H	H···*A*	*D*···*A*	*D*—H···*A*
C9—H9···N1^i^	0.95	2.53	3.377 (2)	149
C3—H3*C*···O2^ii^	0.98	2.65	3.389 (2)	133

Symmetry codes: (i) *x*−1, *y*, *z*; (ii) −*x*+1, *y*+1/2, −*z*+1.
